# Understanding the rationales and information environments for early, late, and nonadopters of the COVID-19 vaccine

**DOI:** 10.1038/s41541-024-00962-5

**Published:** 2024-09-14

**Authors:** Lisa Singh, Le Bao, Leticia Bode, Ceren Budak, Josh Pasek, Trivellore Raghunathan, Michael Traugott, Yanchen Wang, Nathan Wycoff

**Affiliations:** 1 Georgetown University, 37th & O Streets, Washington, DC 20057 USA; 2 University of Michigan, 500 South State Street, Ann Arbor, MI 48109 USA

**Keywords:** Epidemiology, Risk factors

## Abstract

Anti-vaccine sentiment during the COVID-19 pandemic grew at an alarming rate, leaving much to understand about the relationship between people’s vaccination status and the information they were exposed to. This study investigated the relationship between vaccine behavior, decision rationales, and information exposure on social media over time. Using a cohort study that consisted of a nationally representative survey of American adults, three subpopulations (early adopters, late adopters, and nonadopters) were analyzed through a combination of statistical analysis, network analysis, and semi-supervised topic modeling. The main reasons Americans reported choosing to get vaccinated were safety and health. However, work requirements and travel were more important for late adopters than early adopters (95% CI on OR of [0.121, 0.453]). While late adopters’ and nonadopters’ primary reason for not getting vaccinated was it being too early, late adopters also mentioned safety issues more often and nonadopters mentioned government distrust (95% CI on OR of [0.125, 0.763]). Among those who shared Twitter/X accounts, early adopters and nonadopters followed a larger fraction of highly partisan political accounts compared to late adopters, and late adopters were exposed to more neutral and pro-vaccine messaging than nonadopters. Together, these findings suggest that the decision-making process and the information environments of these subpopulations have notable differences, and any online vaccination campaigns need to consider these differences when attempting to provide accurate vaccine information to all three subpopulations.

## Introduction

COVID-19 remains an ongoing public health concern, and all indications suggest that the virus will persist for the foreseeable future^1^. Vaccines have been crucial in mitigating COVID-19, and their uptake as new boosters develop will remain an important element of a public health strategy to manage the continued presence of this virus^2^. Most Americans (81% according to the CDC) have received at least one dose of a COVID-19 vaccine since the shots became widely available in early 2021. While many were initially eager to roll up their sleeves, a moderate portion of the country expressed hesitancy^3^, with some waiting months before getting vaccinated. Around one in five Americans remained unvaccinated two years later^4^, and only one quarter of those who received the original vaccine received the latest recommended booster^5^. Compared to other countries, Americans had lower COVID-19 vaccine acceptance rates (57%) during the initial rollout phase at the end of 2020^6^.

COVID-19 vaccine hesitancy has been associated with political and social divides, questions around safety of vaccines, declining public trust in government and science, and misinformation more broadly^7–16^. In general, online information consumption has been linked to offline behaviors^17^, and there is also evidence that misinformation on the internet, particularly on social media and YouTube, is associated with hesitancy^18^. This presence of online misinformation can be more impactful because it exists against a backdrop of low health literacy with respect to COVID-19^19^. Some research also raised the potential that these sources of misinformation may be associated with partisanship^20–22^. Misinformation about COVID-19 is not limited to the United States; it is a global phenomenon^23^ with false information disseminating worldwide on social media platforms^15,24–29^.

Despite the considerable body of research on vaccine acceptance and hesitancy, there is a gap in our understanding of the more nuanced nature of individual perceptions and decisions about vaccination. Existing work sheds light on two components of this process—the types of people that report hesitancy^30–32^ and the extent to which various message streams contain problematic information^33–35^—but it does not directly link these components, meaning that it is unclear whether certain types of beliefs and attitudes undergird hesitancy or whether both just happen to be present (perhaps because hesitant individuals bolster their rejection of the vaccination by accepting misinformation^36^). To the extent that digital platforms have been acknowledged as a significant source of vaccine information^37–40^, there is a need for a more in-depth examination of how individuals engage with these channels and whether this is related to how they subsequently form their perceptions about vaccination.

This article is an important step toward understanding this relationship. We first determined what factors are associated with decisions to obtain the COVID-19 vaccine. Using the subpopulation categorization presented by Kang et al.^41^, we then compared rationalizations of those who vaccinated early (early adopters), those who initially expressed skepticism about getting vaccinated but later change their minds (late adopters), and those who chose not to get vaccinated during our study period (nonadopters). One important part of understanding vaccine hesitancy is identifying the types of information different subpopulations are exposed to and how that may have influenced their behavior. A number of studies documented high levels of ideological segregation of political information on social media^42–45^. Given this, our final analysis investigated differences in the information environments of these three subpopulations on Twitter/X. This line of inquiry allowed us to better understand any distinguishing features of each of these subpopulations, including the vaccine messaging received and prominent accounts followed.

While previous research investigated the reasons why some people chose to vaccinate and others chose not to^11,46,47^, these studies focused primarily on one particular group (e.g., late adopters^46,47^ or nonadopters^11^). In this longitudinal study, we compared the reasons why early adopters and late adopters chose to get vaccinated, as well as the reasons why the late adopters initially hesitated and nonadopters did not. While investigating all three subgroups, we focused more on those who initially chose not to get vaccinated, but eventually did, in order to gain insight into what the people who changed their minds were thinking and how their rationale aligned with (or deviated from) the other subgroups. This line of research is important because persuasion of initially hesitant individuals constitutes a primary goal for public health officials, and insights into the thought processes and information environments of those who have already undergone this change directly supports this goal. Also, decisions to delay or refuse vaccines have important implications beyond their ability to save lives during the pandemic^48^, including the tendency to stay up to date on subsequent COVID-19 shots and vaccines for other diseases^49^.

## Results

**Fig. 1:**
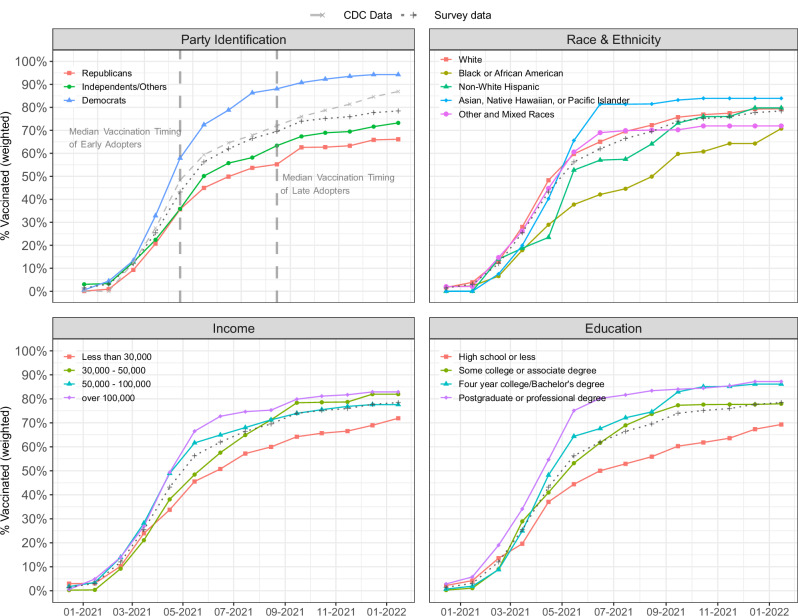
Cumulative vaccination by demographic groups. Each subfigure highlights a different demographic subgroup. The x-axis shows the date of vaccination, and the y-axis shows the overall percent vaccinated for the subgroup. The dark gray dotted line represents the estimates of population vaccination percentages based on our overall survey population. The light gray dashed line represents the vaccination percentages (population 18 and above) based on CDC data. The two vertical lines in the top left subfigure indicate the median vaccination timing for early adopters (left vertical line) and late adopters (right vertical line).

We begin by investigating the rate and time of vaccinations by demographics. Despite various efforts to incentivize vaccination, both the rate and timing of vaccinations varied by partisanship and socioeconomic factors (see Fig. 1). Based on our survey demographics, partisanship explained the most variation, with Democrats getting vaccinated faster than the average CDC reported percentages. Democrats also had significantly higher percentages of vaccination when compared to Republicans and Independents. Within our sample, vaccine percentages were lowest among Black Americans, those with lower incomes (less than $30,000), and those with less education (a high school education or less). All these subgroups also vaccinated at percentages slower than the CDC overall vaccination percentages. Our results also suggested that female, Black, and Hispanic respondents were less likely to get vaccinated early (late adopters), while people in the 45–65 age group, with a postgraduate degree, and Independents and Democrats (compared to Republicans) were more likely to be early adopters. Focusing on those who were initially hesitant (late adopters), we found that they were more likely to have a college degree (OR 1.869 with 95% CI [0.909, 3.945]) and less likely to be parents (OR 0.393 with 95% CI [0.177, 0.846]). (See Supplementary Table 1 for detailed sociodemographic information.) Finally, we found that the median difference in timing between early adopters and late adopters was three and a half months. These findings are consistent with previous literature^20,50,51^.

### Why people do and do not get vaccinated

In addition to the partisan and demographic differences, we are interested in understanding the rationales of the early and the late adopters for getting vaccinated, and the rationales of the late adopters and the nonadopters for not getting vaccinated when the vaccines first became available.

**Fig. 2:**
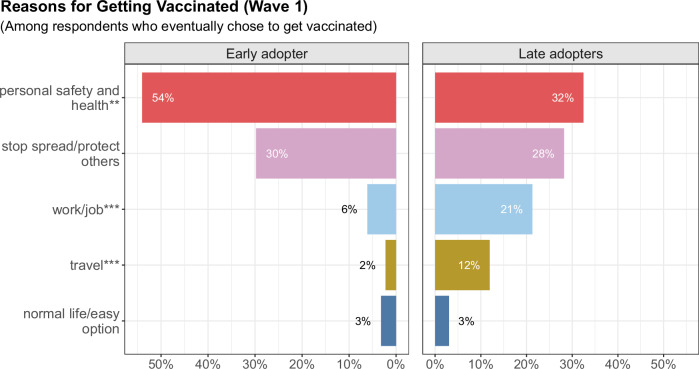
Top 5 open-ended topics on why respondents get vaccinated. The x-axis shows the percentage of responses associated with a specific topic for early adopters (left subfigure) and late adopters (right subfigure). *p < 0.05, **p < 0.01, ***p < 0.001 indicates the topic is statistically significant as a predictor of vaccine status. These topic data are limited to responses from wave 1, as this was the period when both early adopters and late adopters provided their main reasons for getting vaccinated. We focus here on the top 5 out of 10 topics as they make up over 95% of the responses. See Supplemental Materials for exact survey question, full results, and sample topic words.

The top two reasons people chose to get vaccinated were their personal safety and health, and protecting others/stopping the spread of COVID-19 (see Fig. 2). In general, when late adopters did get vaccinated, their top five reasons for doing so were similar to early adopters. However, a smaller proportion of late adopters (32%) cited personal safety and health as their reason for vaccination compared to early adopters (54%); a larger percentage indicated that they chose to get vaccinated because of work/job requirements (21%) and travel (12%), in contrast to early adopters, for whom these reasons were less prevalent at 6% and 2%, respectively. We note that the wave 1 survey occurred when vaccine mandates were being implemented in the federal government and some schools^52^.

**Fig. 3:**
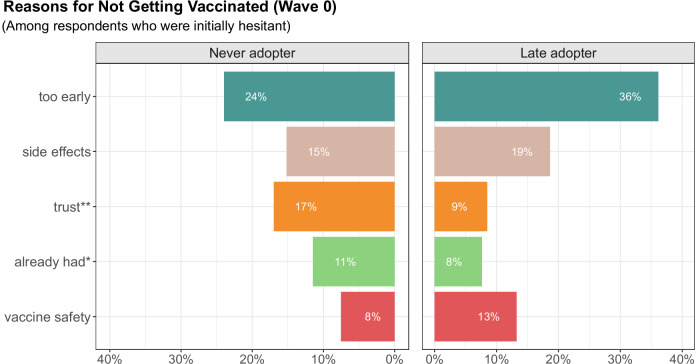
Top 5 open-ended topics explaining why respondents did not intend to get vaccinated. We compare the nonadopters (left side of figure) to the late adopters (right side of figure). These are the top five reasons based on the open-ended responses as to why they chose not to get vaccinated. *p < 0.05, **p < 0.01, ***p < 0.001 indicates the topic is statistically significant at the respective level as a predictor of vaccine status. The topic data are limited to responses from wave 0, as this was the period when both nonadopters and late adopters provided their main reasons for not getting a coronavirus vaccine. We focus on the top 5 out of 14 topics since they make up nearly 80% of the responses. See Supplementary Materials for exact survey question, full results, and sample topic words.

While most of the respondents who did not plan to get vaccinated in March 2021 continued to remain unvaccinated throughout the study period, some respondents changed their mind, getting the recommended shots (between survey waves 0 and 1). Figure 3 shows that the main reason for both late adopters and nonadopters not initially getting vaccinated was because they considered it too early to get the vaccine. This reason was reported by 36% of late adopters, compared to 24% of nonadopters. There were also differences in the percentages of respondents statinglack of institutional distrust and having already had COVID-19 as the reason for not getting vaccinated. Only 9% of the late adopters cited lack of institutional trust as their reason for not getting vaccinated, while it was more prevalent among nonadopters at 17%. In addition, a previous COVID-19 infection was a reason for 8% of late adopters not getting vaccinated compared to 11% of nonadopters. We also found that those who initially mention institutional trust and having natural immunity as reasons for not getting vaccinated were also less likely to get vaccinated later (OR 0.322 with 95% CI [0.125,0.763] and OR 0.281 with CI [0.094,0.760], respectively). (See Supplementary Table 2 for more details.)

When comparing the late adopters and early adopters to explain the timing of vaccine uptake, those who mention personal safety and health as their reasons for getting vaccinated were also more likely to be early adopters; and those mentioning the topic work/job reasons were less likely to get the vaccine early. (See Supplementary Table 3 for more details.)

### Information exposure of Twitter/X users

**Fig. 4:**
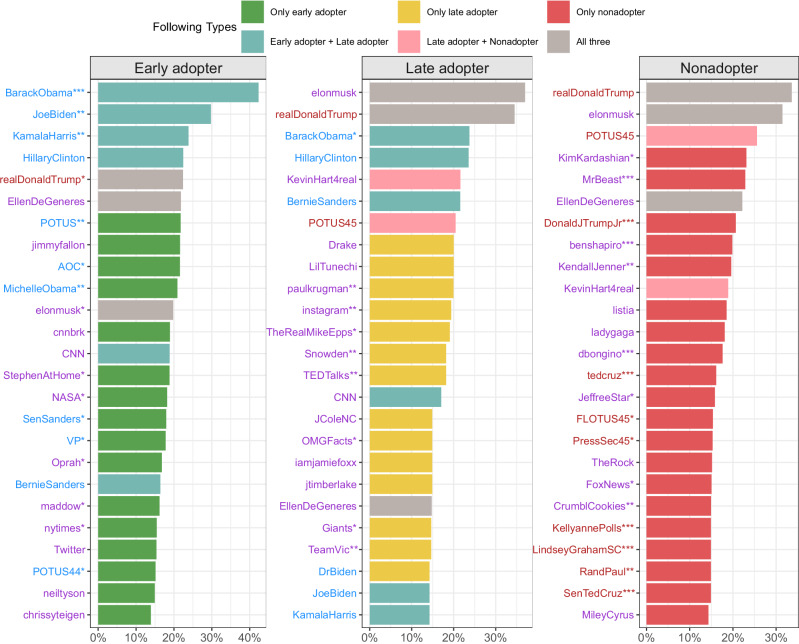
Top 25 followed Twitter/X accounts by vaccine behavior (% of consented Twitter/X respondents from each category: early adopters, late adopters, and nonadopters). The x-axis shows the proportion of consented Twitter/X respondents in that group following each of the prominent accounts listed on the y-axis. The colors of bars show the groups of respondents with different types of vaccine behavior who follow the prominent account. The colors of the labels on the y-axis represent the partisanship of the accounts (blue: Democrats, red: Republicans, purple: apolitical). *p < 0.05, **p < 0.01, ***p < 0.001 indicates the account is statistically significant at the respective level as a predictor of vaccine status.

To consider how information flows might play a role, we turn to participants’ social media, in particular Twitter/X. Among survey respondents who shared Twitter/X accounts, we compared who early adopters, late adopters, and nonadopters each follow on Twitter/X, focusing on *prominent accounts*–the 25 Twitter/X accounts that were followed by the most users in each group (See Fig. 4). We found that there was little overlap in the accounts most frequently followed across all three groups (5% of accounts overlap). Donald Trump, Elon Musk, and Ellen DeGeneres were the only prominent individuals in the top-25 followed accounts who overlapped across all three groups. There was a strong following of mostly Democratic political elites accounts among early adopters and entirely-Republican elites accounts among nonadopters. In general, following politicians’ accounts were the most commonly identified statistically significant factor for predicting vaccine status among early adopters and nonadopters. In contrast, late adopters followed fewer Democratic leaning political accounts than early adopters and fewer Republican leaning political accounts than nonadopters. The political accounts they followed belonged to national leaders of both parties (Trump, Obama, Clinton, Sanders are all in the top 10). Generally, they followed a more diverse set of account types than the early adopters and the nonadopters. The accounts that were most statistically significant predictors of eventual vaccine status among those who were initially skeptical were, for example, an economist’s account (Paul Krugman), a social media platform (Instagram), an educational account (TED Talks), a conspiracy figure’s account (Snowden), and a sports figure’s account (Victor Cruz). In contrast, the best predictors of eventual vaccine status among early adopters were overwhelmingly political accounts.

When we broadened our analysis to the top 100 accounts followed by each group, we obtained a little more nuanced picture. The largest account type followed by our consented Twitter/X respondents was entertainment with nonadopters following them at the highest percentage (52%) comparing to early adopters (39%) and late adopters (45%). Early adopters follow more political and news accounts (43%) compared to the other two groups (31% for late adopters and 30% for nonadopters), and late adopters tended to follow slightly more science/tech accounts (12%) compared to early adopters (7.5%) and nonadopters (6%). (See Supplementary Fig. 5 for details about the types of accounts being followed by out three groups).

**Fig. 5:**
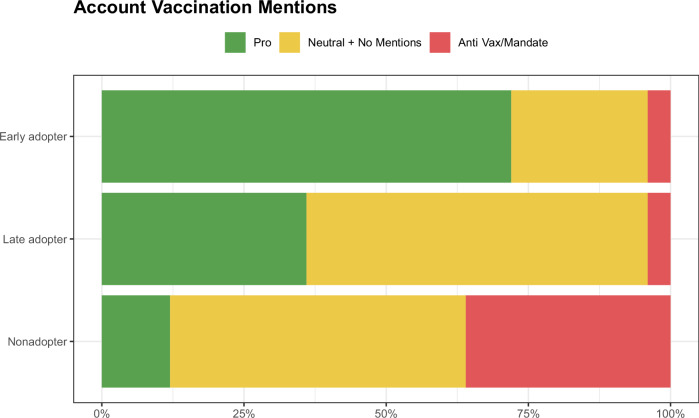
Vaccine mention by the top 25 prominent accounts. The x-axis shows the proportion of consented Twitter/X respondents in a specific group exposed to different vaccine-related messaging. Green represents the proportion of prominent accounts that shared pro-vaccination messaging (Pro). Red represents the proportion of prominent accounts that shared anti-vaccination or anti-vaccine mandate messaging (Anti Vax/Mandate). Yellow represents the proportion of prominent accounts that shared neutral vaccination messaging or no vaccination messaging (Neutral + No Mentions). There are no prominent accounts that shared both pro and anti-vaccine messaging.

Next, we looked only at the vaccine-related content of the previously identified prominent accounts. Figure 5 shows the distribution of different message types for our set of prominent accounts. We see that the prominent accounts followed by early adopters shared a high amount of pro-vaccination messaging (72% of accounts), while the prominent accounts followed by late adopters shared a more modest level of pro-vaccination messaging (36% of accounts). In contrast, based on the accounts they followed, nonadopters heard anti-vaccination or anti-vaccine mandate messaging significantly more than the other two groups combined (36% of accounts as opposed to 8%) and pro-vaccination messaging less than each of the other two groups (12% as opposed to 72% for early adopter and 52% for late adopters). Early adopters were over five times more likely than nonadopters to have heard pro-vaccination messaging from prominent accounts, while late adopters were three times more likely.

**Fig. 6:**
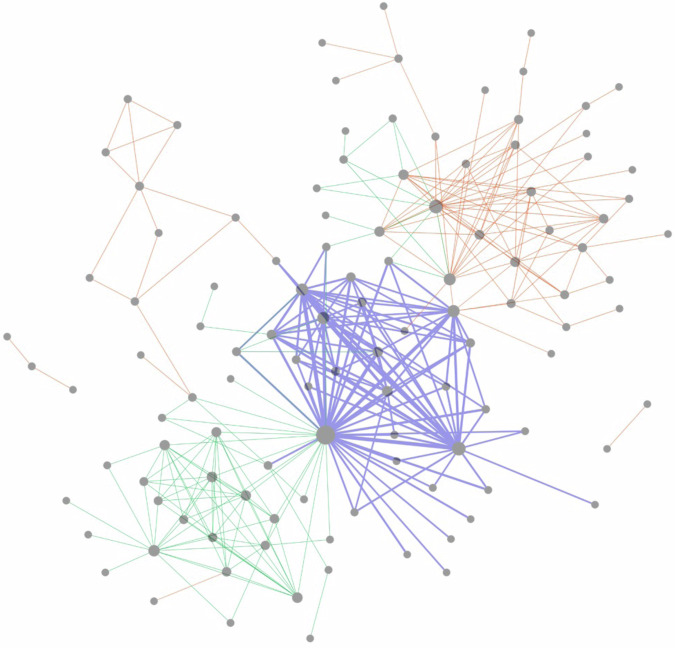
Network diagram of accounts followed by respondents on Twitter/X. Nodes represent prominent accounts followed by consented Twitter/X respondents. An edge indicates that at least two respondents follow the pair of accounts. The thickness of the edge is based on the number of respondents following different pairs of nodes. The edge color shows the respondents vaccine status (purple = early adopter, green = late adopter, red = nonadopter). In cases where multiple edges exist between a pair of nodes, we show a single edge with an edge coloring determined using edge weight. There are only four cases when multiple edges occur between a pair of nodes. The four nodes with 2 edges between them are the following (DonaldJTrumpJr, realDonaldTrump), (DrBiden, BarackObama), (DrBiden, JoeBiden), and (elonMusk, realDonaldTrump). See Supplementary Fig. 1 for a labeled version of the network.

Evidence that individuals with certain beliefs and behaviors followed particular sets of accounts could be a product either of the specific information those accounts share or the kinds of networks in which those accounts are embedded. Although we found suggestive evidence that the vaccine content of the individuals being followed differed across groups, it is still important to understand what those information networks looked like on an individual level. To better understand the connections across accounts that were followed, Fig. 6 shows a network that highlights the relationship between prominent accounts followed by our respondents in the consented Twitter/X population (See Supplementary Fig. 1 for the labeled version of network). The average degree and weighted degree of the network were 5.18 and 78.14, respectively. There were three connected components and the largest had a diameter of 9. We found that there are clear distinct clusters for each vaccine status group, particularly the nonadopters and the early adopters. The modularity was 0.18 and the average clustering coefficient was 0.41, confirming the network’s clear group structure. The highest degree (largest) nodes in the purple cluster (early adopters) were the handles BarackObama, POTUS, and JoeBiden. The highest degree nodes in the red cluster (nonadopters) were the handles realDonaldTrump and ElonMusk. The green cluster (late adopters) did not contain nodes with as high a degree as the other clusters. Its highest degree node was handle CFBPlayoffs.

Our late adopters shared more network nodes with both groups than the two groups shared with each other. They shared 18 edges with early adopters and 13 edges with nonadopters. Among those not intending to vaccinate or unsure in the first wave, when predicting eventual vaccination status based on high-ranking Twitter/X followers, demographics, and survey response topics, a combination of Twitter/X follows and demographics was most informative, with a predictive cross entropy of approximately 0.45 on average compared to a predictive cross entropy of 0.5 for demographics alone or Twitter/X follows alone (*p* < 0.001, See Supplementary Fig. 2).

We also found that across all network metrics except the number of connected components, differences in the metrics determined from our graph compared to those generated using random graphs were statistically significant. For example, the clustering coefficient has 95% CI [0.0252, 0.0682] and the largest diameter has 95% CI^5,7^ (see Supplementary Table 4 for more details). In other words, the connectivity structure and clustering behavior were not random.

## Discussion

Our demographic results about vaccine hesitancy are consistent with previous research^20,50,51^. The panel design of our study and the coupling of social media and survey analysis allows us to investigate what factors lead initially hesitant respondents to eventually vaccinate (late adopters), and gives insight into prominent information sources they follow on Twitter/X. This leads to our unique findings differentiating this group from early adopters and nonadopters (differences in rationales and in Twitter/X information they are exposed to). While the reasons that people are initially hesitant overlap, our results suggest that vaccine mandates are an important strategy for persuading late adopters to get vaccinated, in addition to the desire to travel and return to a more normal lifestyle. While these matter to early adopters, they are less important. When comparing late adopters and nonadopters, the primary reason of it being too early was the same for both subgroups. However, the differences in the proportion of respondents stating institutional distrust as a reason for not getting vaccinated was significant with nonadopters mentioning it almost twice as often. While these top reasons align with the previous findings indicating that lack of government trust is a primary reason against vaccination^11^, as well as echoes of tropes anti-vaxxers use^53^, the divergence between nonadopters and late adopters is crucial, with the latter group reporting rationales that are more temporal in nature. This implies that they may have always been more open to changing their stance on vaccination once safety concerns were alleviated.

In addition, the vaccine-related information obtained on Twitter/X by our three subpopulations differed. Early adopters follow a large number of prominent politicians who are Democrats, and also see a large percentage of pro-vaccine messages. The nonadopters in our sample, on the other hand, follow a large number of prominent politicians who are Republican, and were potentially exposed to five times more anti-vaccine and/or anti-vaccine mandate messaging than the other two groups. The late adopters are in the middle of these two extremes - while they are exposed to some anti-vaccination messaging, they also follow prominent politicians who share pro-vaccine messages and follow many prominent non-politicians, most of whom either do not post about vaccines at all, or post support for them. In general, they follow a diverse set of accounts, many of which post support for vaccinations. From our network analysis, we see that clear clusters emerge based on who respondents in different vaccination groups follow. Similar to Johnson and colleagues’ study of Facebook users, we find that late adopters are connected to prominent accounts that are both pro-vaccine and anti-vaccine, creating messaging competition^54^. However, in our sample, the proportion of pro-vaccination messaging is significantly higher than anti-vaccination messaging within both the early adopter and late adopter groups.

Both the demographic results and the Twitter/X followers analysis descriptively support our statistical analysis. In both cases a combination of demographic factors and Twitter/X follows is most informative for predicting the likelihood of vaccinating. Our research design does not allow us to determine whether social media information environments led to changes in vaccine attitude or whether this rich information is simply a correlate. But the value of social media data is clear. Whether a cause or correlate, such data can help us identify vaccine attitudes more accurately than is possible using only demographics data alone. In short, what seems to be the primary difference between those who eventually choose to be vaccinated and those who remain unvaccinated are 1) the reinforcement of anti-vaccination messaging from prominent individuals and 2) a general distrust in government.

Social media platforms have increasingly become the primary source of news and information for many people. Although it is not the modal experience^55^, individuals on these platforms can exist in *echo chambers*, where they predominantly follow, consume, trust, and share content that mirrors their existing beliefs^56–59^. This reinforcement loop means users are more likely to interact with, and perhaps be influenced by, content and individuals that affirm their pre-existing views. This trend can be amplified during times of uncertainty, e.g., during a pandemic. Prior research has shown that social media can impact beliefs and outcomes related to vaccination^40^, and this impact is more pronounced when individuals receive information from trusted in-group sources^60,61^. Both early adopters and nonadopters on Twitter/X seem to have more significant exposure to prominent accounts that already align with their existing political stands. In contrast, even though late adopters follow political accounts, they generally follow a more diverse range of prominent accounts on social media and are likely exposed to a wider array of high- and low-quality information. If we view early adopters and nonadopters as firmly locked in their political camps and respective views on vaccination, the late adopters represent the “ambiguous public” -- a middle group potentially open to persuasion^62,63^. This highlights the importance of both trust and information quality in the age of misinformation. The role of trusted political and non-political figures in setting the initial position and subsequently impacting people’s behavior is evident and crucial. While our experimental frame does not allow us to establish causality, the existence of a relationship between online messaging and behavior is clear.

Finally, our analysis further confirms previous studies that highlight how prevalent and problematic a growing lack of trust in government may be^9,11,64–68^. In this sample, nonadopters cited distrust as one of the major reasons for not getting vaccinated. This was an important difference between those who eventually received the vaccine and those who did not. However, when we look at our Twitter/X sample, we see that both early adopters and nonadopters follow a similar number of prominent politicians, with early adopters following more Democrats and nonadopters following more Republicans. We conjecture that the decline of public trust in government is associated with the growing anti-establishment sentiment. This trend is particularly pronounced with the rise of anti-establishment politicians in the United States like Donald Trump and Rand Paul, and opinion leaders such as Elon Musk. Studies find that anti-establishmentarianism is associated with antisocial psychological traits, the acceptance of political violence, time spent on extremist social media platforms, support for populist politicians, and belief in misinformation and conspiracy theories^69,70^. Ultimately, if anti-establishment leaders (political and otherwise) advocate an anti-vaccination campaign online, it will continue to impact vaccine acceptance rates.

The primary limitation of this study is its observational nature: it is not possible to disentangle two scenarios related to social media. The first possible scenario is that individuals are being influenced by the content of their Twitter/X feeds and are being moved to change their vaccination behavior as a result. The second possible scenario is that, having already solidified their vaccination intentions, individuals then follow accounts on Twitter/X that reinforce their existing viewpoints and validate their previously made decisions. We expect reality to be somewhere in the middle. This makes it difficult to estimate with certainty the effectiveness of an intervention deployed via social media. But regardless of the extent of these two effects, knowing which accounts are associated with vaccination hesitancy is a useful barometer of public sentiment, as information about who follows which accounts is easily accessible.

Additionally, due to individual differences in platform usage and to the nature of social media algorithmic curation we do not know how often the respondents access Twitter/X, or what specific information they see when they do. We only know how often they post or repost content. Furthermore, Twitter/X is only one of many sources of information respondents are exposed to. Other sources include traditional media and personal networks. Any given individual may be more or less reliant on Twitter/X as a source of information, and the persuasiveness of the information they encounter there also likely varies on an individual basis.

There are also the standard limitations associated with any survey-based study that requires additional consent. Namely, there can be bias associated with survey non-response, as well as associated with consenting to share and link the Twitter/X account information^71^. As we have seen, a lack of trust in institutions is associated with vaccine hesitancy, and it is plausible that this may also be associated with a reduced willingness to participate in research in general and to share social media profiles more specifically^72^. Additionally, there is a risk that some respondents may misreport their vaccination status as a function of social desirability and that their responses to the open-ended questions may not reflect the true reasons that individuals behaved the way they do – if indeed they can even identify such reasons.

Moving forward, these findings highlight the importance of using both social media and traditional media to inform the public about the need to vaccinate and the safety of vaccines. Reinforcement of the need to obtain vaccinations by prominent social media accounts (including those of entertainment, news, science, and sports figures) could be important for those who are hesitant but persuadable because prominent accounts may be viewed by the public as trusted surrogates when information is hard to access or polluted by misinformation. New strategies should be explored to engage different types of social media influencers and personalities in vaccine messaging. The recent campaign featuring Travis Kelce encouraging the public to get COVID-19 and flu vaccines simultaneously is a great example of a cross-media campaign that could be effective in persuading skeptics^73^. Vaccine messaging that is more randomly shared through advertising may be useful for those who are already likely to get vaccinated (early adopters), but posts shared by influencers may be more beneficial for those who are less inclined to get vaccinated (late adopters). We also suggest regular public announcements on different social media and traditional media platforms that focus on vaccine safety more broadly, as well as announcing the importance of vaccinations through stories of how vaccines have saved lives. Storytelling is a particularly effective persuasive device^74^. Continual exposure to pro-vaccine messaging is vital to decreasing the impact of poor-quality vaccine-related information and increasing health literacy. Recall our finding that individuals who are initially skeptical but eventually received a vaccine (late adopters) are most concerned about side effects of the vaccine, suggesting that messaging about the safety of the vaccine may help alleviate some of these concerns and lead to higher rates of vaccinations. If public health officials do not actively share and reinforce the health benefits of vaccinations and how effective and safe they are, the information pollution generated by anti-vaxxers will likely continue to increase vaccine hesitancy.

Conversely, our finding that the greatest concern among those who remain unvaccinated is trust in institutions implies that trust-building efforts to date have not had their desired effects. Trust-building is also not a quick process that can be undertaken in response to a crisis. Increasing trust in government and reducing the politicization of vaccinations will require persistent effort if we want better outcomes during the next public health emergency.

The primary question left open by this article is that of causality between social media exposure and vaccination, but this study makes it easier to tackle that question in future work. The most robust research design would involve a controlled experiment where subjects modify their social media behavior. Some such studies ask the user not to use social media at all (see e.g., ref. ^75^). Another avenue of experimentation consists of exposing subjects to particular social media posts^76^, and then asking the subjects attitudinal questions and comparing the responses with those of a control group. Some previous research has even used platform-based manipulations to assess social media impacts^77^. Our results motivate such an experiment, exposing subjects to messaging from political elites, both Republican and Democratic, and measuring whether this hardens attitudes on vaccines relative to a control group. Similarly, one can ask subjects to subscribe to these same accounts and rely on the social media platform to expose them to the messaging^78^ or, in collaboration with the platform, alter the extent to which certain users receive messages from certain accounts^79^.

There are also many other important future directions. One direction is to investigate other social media platforms, and ideally, to simultaneously look at multiple platforms. Additionally, communications from employers or health insurance companies and government entities may have played a role in decision making. Capturing that information in a survey or from health providers is also a fruitful direction. Furthermore, personal offline networks are likely to play a moderating role in vaccination behavior. To investigate this, we could imagine either simply adding questions about personal networks in a survey much like the one conducted in this study, or even using a more sophisticated survey design, such as snowball sampling, which would allow researchers to collect information from respondents who know one another personally to investigate correlations in behavior not explained by social media activity. Finally, all of the information on social media occurs against the backdrop of traditional media, government messaging, and other sources of information. Future work could incorporate both traditional and non-traditional information sources.

In conclusion, we find that the self-reported rationalizations for vaccine hesitancy differs among those who eventually decide to be vaccinated and those who choose not to. There is also a difference in terms of the types of accounts followed by each group and the amount of pro- versus anti-vaccine messaging our Twitter/X subsample was exposed to. It is also important to note that nonadopters tend to follow prominent Republican political accounts and express a lack of trust in institutions. By contrast, late adopters are less likely to follow only prominent political accounts and are more motivated by a desire to return to normal life and work requirements to be vaccinated. Our findings also suggest that we must engage in strategies to increase trust in government. Addressing the fundamental issues that cause a lack of trust in institutions is a whole-of-society problem. Research has noted that Americans’ trust in government, in particular the national government, has declined over the past half century^80^. Especially in the age of misinformation and during critical times like pandemics, trust in government becomes even more vital for cohesive societal responses. Other important strategies include improving the digital experience for those who engage with government, increasing capacity to effectively deliver public services, and sharing success stories and narratives of public servants on social media and other platforms. Ultimately, the battle for increasing vaccination uptick includes a secondary battle to improve trust in government.

## Methods

We used micro-linked data from a panel survey and Twitter/X (i.e., closed-ended survey response, open-ended text data, social media post text data, and network data) and implemented a series of analytical techniques, including topic modeling, statistical models, and network analysis to gain a more comprehensive and nuanced understanding of respondents’ attitudes toward COVID-19 vaccines.

### Consent

All survey participants consented to participation in the research via online forms. More details in Section IV.1.

### Ethical approvals

This study obtained IRB approval. The study was approved via Georgetown University IRB under protocol STUDY00003571.

### Micro-linked data sample

We leveraged the SSRS Opinion Panel for survey recruitment (*N* = 9544), a probability-based panel of U.S. adults ages 18 or older and recruited randomly based on a nationally representative ABS (Address Based Sample) probability design (including Hawaii and Alaska)^81^. Survey recruitment was conducted via the web in three waves (Wave 0: March 1 to June 15, 2021; Wave 1: October 11 to October 20, 2021; Wave 2: January 27 to February 9, 2022) and included survey questions on the SSRS Opinion Panel registration survey for new panelists as well as survey questions on demographic refreshment surveys conducted among existing SSRS Opinion Panel members. The overall cumulative response rate (AAPOR – RR3) was 4% for wave 0, the full panel sample. Among a recruited sample of 9544 panelists, 9468 were interviewed in English and 76 in Spanish. Data were weighted to adjust for ABS recruitment, and raking weights were produced to represent the U.S. adult population.

Wave 1 and 2 were samples of Wave 0. For Wave 1, a subset of the SSRS Opinion Panel participated in new data collection via the web from October 11 – 20, 2021, resulting in a sample of 1003 participants. For Wave 2, a subset of the SSRS Opinion Panel participated in new data collection via the web from January 27- February 9, 2022, resulting in a sample of 1000 participants. In each wave, data were weighted to represent the target U.S. adult population. These weighted survey responses were used to generate descriptive and statistical results. Respondents were asked via multiple choice questions to self-report their vaccination intentions (Wave 0) or behavior (Waves 1 and 2), as well as for the date during which they received their vaccination if applicable. Our statistical analyses primarily focused on respondents who participated in both Wave 0 and Wave 1 of the study. This is to maximize the number of respondents included in the analysis, for whom we can consistently track observed behavioral changes and topic mentions within the same time period. Wave 2 was primarily used to determine vaccine uptick percentages through time for different demographic groups. Among 1532 respondents who participated in at least one follow-up wave of surveys, 9 respondents mentioned they planned to get vaccinated but never did by the time of their final survey response. We excluded this group from the analysis.

Among 9544 survey participants in Wave 1, 3140 were Twitter/X users, and among these, 735 were willing to provide their handle. For a detailed assessment of potential consent bias, Supplementary Figs. 4 and 5 provide systematic comparisons between Twitter/X users and consenting survey respondents. We looked at posts shared by respondents between March 1, 2021, and February 28, 2022. We also collected the accounts that the consented Twitter/X respondents were following and used these accounts as proxies for information exposure and sources. We selected Twitter/X as our primary platform for several reasons. First, the Twitter/X posts are expected to be visible publicly, aligning with our considerations of privacy and ethics. Second, its wide use in the U.S. with over 20% of Americans actively using the platform at the time of our study means that a reasonable portion of the population or at least an important subpopulation consume information and communicate online through this platform. Additionally, the process of collecting Twitter handles from survey respondents proved to be relatively straightforward (compared to other platforms), as users typically recall their handles easily when completing surveys.

### Topic modeling

In order to quantitatively evaluate the open-ended question responses, the exact responses to open-ended questions were transcribed by interviewers and coded using a semi-supervised guided topic model, GTM^82^. Preprocessing steps included capitalization standardization, punctuation removal, and stopword removal. Frequently occurring words and phrases were identified by counting the frequency with which respondents used different unigrams, bigrams, and trigrams. In order to generate an initial set of topics for each open-ended response, researchers on our team looked through the list of frequently occurring words and phrases to identify those that could be combined to form specific topics. These initial topics were inputs into GTM^82^. The model produced additional words for each identified topic and produced new topics. The research team reviewed all the added words and new topics. Topics were manually adjusted and added when at least two out of three researchers agreed on the change. This process was conducted iteratively at least three times for each open-ended question, until the researchers were satisfied with the resulting topic list. Responses were then labeled. This full human-in-the-loop process was repeated until at least 85% of responses were labeled for each open-ended question.

### Statistical analysis

In order to compare the predictive ability of demographic factors, survey responses and Twitter/X follows, we conducted out-of-sample analysis. Specifically, for each of 100 iterations, we randomly held out 20% of the respondents who had consented to Twitter/X linking and used the remaining 80% of such respondents to fit a logistic regression model. We used the following variable groups in our analysis: only demographic information, only Twitter/X follows, only survey data, and a combination of all the variable groups. We then used these models to predict the vaccination status of the remaining 20%. In the first analysis, the response variable is given as one of the three vaccination classes (early adopter, late adopters, or nonadopters) a respondent belongs to, and the model is specified as a multiclass logistic regression (see Supplementary Fig. 3).

We additionally conducted two-class logistic regressions in order to contrast the characteristics of nonadopters from late adopters (Supplementary Table 2), as well as late adopters from early adopters (Supplementary Table 3). In each case, we conducted a regression based only on sociodemographic factors (left column of coefficients), as well as one using both sociodemographics and survey topics (right column).

### Twitter follow and network analysis

We used the Twitter4J software package to access the Twitter/X Timeline API and collected 24,076 accounts followed by at least two consented survey respondents who used Twitter/X. This collection occurred after wave 2. Within the descriptive network analysis, we identified the top 100 commonly followed pairs of accounts for each of our three vaccination status groups. Using the statistical analysis described in the previous subsection, we included the top 25 accounts followed by each of the three outcome groups when estimating the effects of following the account on predicting the vaccination status. For Figs. 4 and 5, we focused only on the most-followed, prominent accounts to identify the common information exposure for each group. For Fig. 5, we considered all vaccine-related content, and then classified it as pro-vaccine, anti-vaccine (including posts against vaccine mandates), or neutral on vaccines. For the analysis of Supplementary Fig. 5, researchers on our team manually classified each of the top 100 accounts into the following categories: Entertainment, Politics (Democratic, Republican, or Nonpartisan), News, Science/Technology, Sports and Others. Each account was classified by at least two researchers and disagreements were discussed among all three.

We also created a uni-modal, multi-edge, weighted network. Nodes represented prominent accounts, and an edge was added between two nodes if at least 2 respondents followed both nodes (accounts). The edge was colored by vaccine status of the respondents following the pair and the width of the edge was based on the number of respondents following the pair. There were only four cases when multiple edges occurred between a pair of nodes. Along with measuring network metrics (weighted degree, betweenness, clustering coefficient, and eigenvector centrality), we determined whether our network was random. We accomplished this by simulating random networks with the same number of nodes and edges as our prominent accounts network. We conducted this simulation 1000 times, determined the network metrics for each constructed network, and reported the average metric value and the confidence intervals.

## Supplementary information

Supplemental Materials

## Data Availability

The survey data for conducting the analysis are available from the authors upon reasonable request. The study protocols and designs are described in the Supplementary Appendices.

## Code availability

The Guided Topic Model (GTM) code can be accessed from Github (https://github.com/GU-DataLab/topic-modeling). The statistical analysis code can also be accessed from Github (https://github.com/georgetown-mdi/vax_adoption).
