# Supplementary material for: Understanding the rationales and information environments for early, late, and nonadopters of the COVID-19 vaccine
Source: NPJ Vaccines. 2024 Sep 14;9:168. doi: 10.1038/s41541-024-00962-5 (PMC11399438; doi:10.1038/s41541-024-00962-5)
Supplement: Supplementary file 1 — Supplemental Materials [file 41541_2024_962_MOESM1_ESM.pdf]

Supplementary Information for
Understanding the rationales and information environments of those who vaccinate early, those who vaccinate later, and those who remain unvaccinated.

Table of Contents

- Page 2: Supplementary Table 1 Demographic Information by Vaccination Status
- Page 3: Supplementary Table 2 Explaining Changes in Vaccine Status by Demographics and Topics (Logit)
- Page 4: Supplementary Table 3 Comparing Early and Late Vaccination by Demographics and Topics (Logit)
- Page 5: Supplementary Figure 1. Labeled network diagram of accounts followed by respondents on Twitter/X
- Page 6: Supplementary Table 4. Network Metrics
- Page 6: Supplementary Table 5. Supplementary List of Open-Ended Topics on Why Respondents Get Vaccinated
- Page 6: Supplementary Table 6. Supplementary List of Open-Ended Topics Explaining Why Respondents Did Not Intend to Get Vaccinated
- Page 7: Supplementary Table 7. Sample Topic Words for Getting Vaccinated Topics
- Page 7: Supplementary Table 8. Sample Topic Words for Not Getting Vaccinated Topics
- Page 8: Supplementary Figure 2. Predictive study estimating vaccination status with various variables.
- Page 9: Survey Questions
- Page 10: Supplementary Figure 3. Twitter Consent Check by Demographics and Vaccine Status.
- Page 11: Supplementary Figure 4. Twitter Consent Check by Open-Ended Flags.
- Page 12: Supplementary Figure 5. Top Followed Accounts by Account Types

Supplementary Table 1. Demographic Information by Vaccination Status.

	Overall	Early Adopters	Late Adopters	Nonadopters
**Count**	1532	1246	104	182
**Gender**				
**Male**	43%	44%	27%	41%
**Female**	56%	54%	72%	58%
**Other**	2%	2%	1%	1%
**Age**				
**[18, 29]**	12%	12%	13%	14%
**[30, 49]**	43%	40%	58%	55%
**[50, 64]**	24%	26%	17%	18%
**[65, inf]**	18%	20%	5%	10%
**Education**				
**High school grad or less**	12%	9%	17%	25%
**Some college**	27%	24%	41%	37%
**College+**	61%	66%	39%	37%
**Income**				
**<30,000**	15%	13%	24%	27%
**30,000-74,999**	33%	32%	39%	32%
**75,000+**	52%	55%	37%	40%
**Home Ownership**				
**Own Home**	71%	74%	57%	59%
**Renting**	29%	26%	43%	41%
**Political Affiliation**				
**Democrat**	45%	51%	31%	9%
**Republican**	19%	14%	33%	43%
**Other**	36%	35%	37%	48%
**Family Status**				
**Married**	54%	54%	48%	54%
**Unmarried**	46%	46%	52%	46%
**Parent**	31%	27%	38%	51%
**Not a Parent**	69%	73%	62%	49%
**Race**				
**Asian**	3%	3%	1%	1%
**Black**	9%	8%	20%	8%
**Hispanic**	12%	11%	16%	12%
**White**	70%	72%	56%	68%
**Other Race**	7%	6%	7%	12%

Note: Underemployed means unemployed or employed part time. These figures consider only the 1,532 who participated in multiple waves, such that their vaccination intentions and status could be determined.

Supplementary Table 2. Explaining Changes in Vaccine Status by Demographics and Topics (Logit)

	Dependent variable:		
	Nonadopter (0) vs. Late Adopter (1)		
	Demographics only	Topics Only	Demographics + Topics
	(1)	(2)	(3)
Female	1.394 (0.888, 2.206)		1.395 (0.850, 2.307)
Age: 30-44	1.092 (0.435, 2.788)		1.181 (0.413, 3.483)
Age: 45-65	0.738 (0.289, 1.885)		0.771 (0.274, 2.204)
Age: 65+ years	0.424 (0.122, 1.402)		0.347 (0.085, 1.327)
Black or African American	1.480 (0.627, 3.472)		0.891 (0.340, 2.293)
Non-White Hispanic	3.079 (1.040, 9.491)		2.227 (0.665, 7.662)
Other and Mixed Race	0.394 (0.059, 1.758)		0.370 (0.047, 2.036)
College	1.385 (0.732, 2.658)		1.869 (0.909, 3.945)
Income: 30,000-50,000	1.152 (0.444, 2.978)		1.457 (0.478, 4.487)
Income: 50,000-100,000	1.231 (0.551, 2.768)		1.304 (0.531, 3.227)
Over 100,000	0.548 (0.223, 1.325)		0.456 (0.158, 1.276)
Married or living with a partner	1.271 (0.615, 2.696)		1.203 (0.536, 2.773)
Parenthood	0.380 (0.186, 0.751)		0.393 (0.177, 0.846)
Employed	1.577 (0.793, 3.171)		1.514 (0.690, 3.369)
Party ID: Independents/Others	0.819 (0.437, 1.536)		0.779 (0.386, 1.570)
Party ID: Democrats	1.682 (0.606, 4.734)		1.690 (0.568, 5.131)
Survey topics: side effects		1.280 (0.653, 2.505)	0.802 (0.331, 1.895)
Survey topics: distrust in science		0.537 (0.094, 2.368)	0.532 (0.077, 2.945)
Survey topics: trust		0.310 (0.132, 0.668)	0.322 (0.125, 0.763)
Survey topics: FDA approval		0.380 (0.043, 1.804)	0.425 (0.037, 3.245)
Survey topics: likely misinformation		0.233 (0.001, 3.444)	0.197 (0.001, 5.456)
Survey topics: too early		0.859 (0.481, 1.521)	1.024 (0.502, 2.076)
Survey topics: already had		0.312 (0.118, 0.738)	0.281 (0.094, 0.760)
Survey topics: most people recover		0.254 (0.044, 0.939)	0.353 (0.053, 1.540)
Survey topics: generally anti-vax		0.916 (0.223, 3.597)	1.501 (0.307, 7.086)
Survey topics: vaccine safety		1.291 (0.592, 2.828)	1.583 (0.580, 4.354)
Survey topics: individual freedom		0.261 (0.031, 1.250)	0.101 (0.002, 0.915)
Survey topics: does not prevent getting covid		0.439 (0.002, 9.330)	0.311 (0.001, 9.063)
Intercept	0.524 (0.170, 1.568)	1.003 (0.643, 1.570)	0.778 (0.211, 2.808)
Observations	254	278	254
Log Likelihood	-132.864	-151.394	-116.467
Akaike Inf. Crit.	299.728	332.787	294.934

Note: Log ratios and associated 95% confidence intervals are reported. The observations are limited to respondents who participated in at least one of the follow-up surveys. The survey topics are based on only wave 0, where all the unvaccinated respondents were asked the open-ended question about the reasons for not getting vaccinated.

Supplementary Table 3. Comparing Early and Late Vaccination by Demographics and Topics (Logit)

	Dependent variable:		
	Late Adopter (0) vs. Early adopter (1)		
	Demographics only	Topics Only	Demographics + Topics
	(1)	(2)	(3)
Female	0.442 (0.254, 0.742)		0.511 (0.279, 0.904)
Age: 30-44	0.643 (0.234, 1.686)		0.428 (0.133, 1.260)
Age: 45-65	4.899 (1.609, 15.630)		4.445 (1.159, 17.946)
Age: 65+ years	4.028 (1.104, 16.546)		2.276 (0.521, 10.565)
Black or African American	0.113 (0.040, 0.317)		0.102 (0.033, 0.311)
Non-White Hispanic	0.221 (0.084, 0.602)		0.273 (0.096, 0.807)
Other and Mixed Race	1.286 (0.294, 11.033)		1.196 (0.226, 13.795)
College	0.910 (0.377, 2.090)		1.128 (0.440, 2.780)
Income: 30,000-50,000	0.825 (0.281, 2.438)		0.691 (0.197, 2.419)
Income: 50,000-100,000	0.840 (0.287, 2.386)		0.758 (0.229, 2.401)
Over 100,000	1.572 (0.413, 6.086)		1.060 (0.228, 4.839)
Married or living with a partner	0.962 (0.413, 2.201)		0.823 (0.317, 2.055)
Parenthood	1.069 (0.467, 2.513)		1.210 (0.489, 3.078)
Employed	0.618 (0.272, 1.362)		0.816 (0.324, 2.025)
Party ID: Independents/Others	5.368 (2.434, 12.300)		4.676 (1.921, 11.821)
Party ID: Democrats	27.536 (9.927, 86.980)		19.311 (6.373, 66.750)
Survey topics: stop spread/protect others		0.791 (0.459, 1.369)	1.429 (0.638, 3.320)
Survey topics: scared/safety/heath		2.170 (1.204, 3.886)	2.187 (0.941, 5.102)
Survey topics: travel		0.208 (0.089, 0.510)	0.693 (0.167, 3.464)
Survey topics: work/job		0.233 (0.121, 0.453)	0.292 (0.112, 0.766)
Survey topics: science		3.428 (0.425, 566.517)	1.631 (0.160, 283.716)
Survey topics: incentive		0.116 (0.010, 1.236)	0.053 (0.003, 0.750)
Intercept	3.765 (1.139, 13.412)	9.113 (5.222, 16.557)	3.671 (0.798, 18.242)
Observations	823	876	823
Log Likelihood	-114.792	-179.671	-99.225
Akaike Inf. Crit.	263.584	381.341	252.449

Note: Log ratios and associated 95% confidence intervals are reported. The observations only include respondents who participated in wave 0 and wave 1. The survey topics are based on only wave 1, where all the vaccinated respondents were asked the open-ended question about the reasons for getting vaccinated.

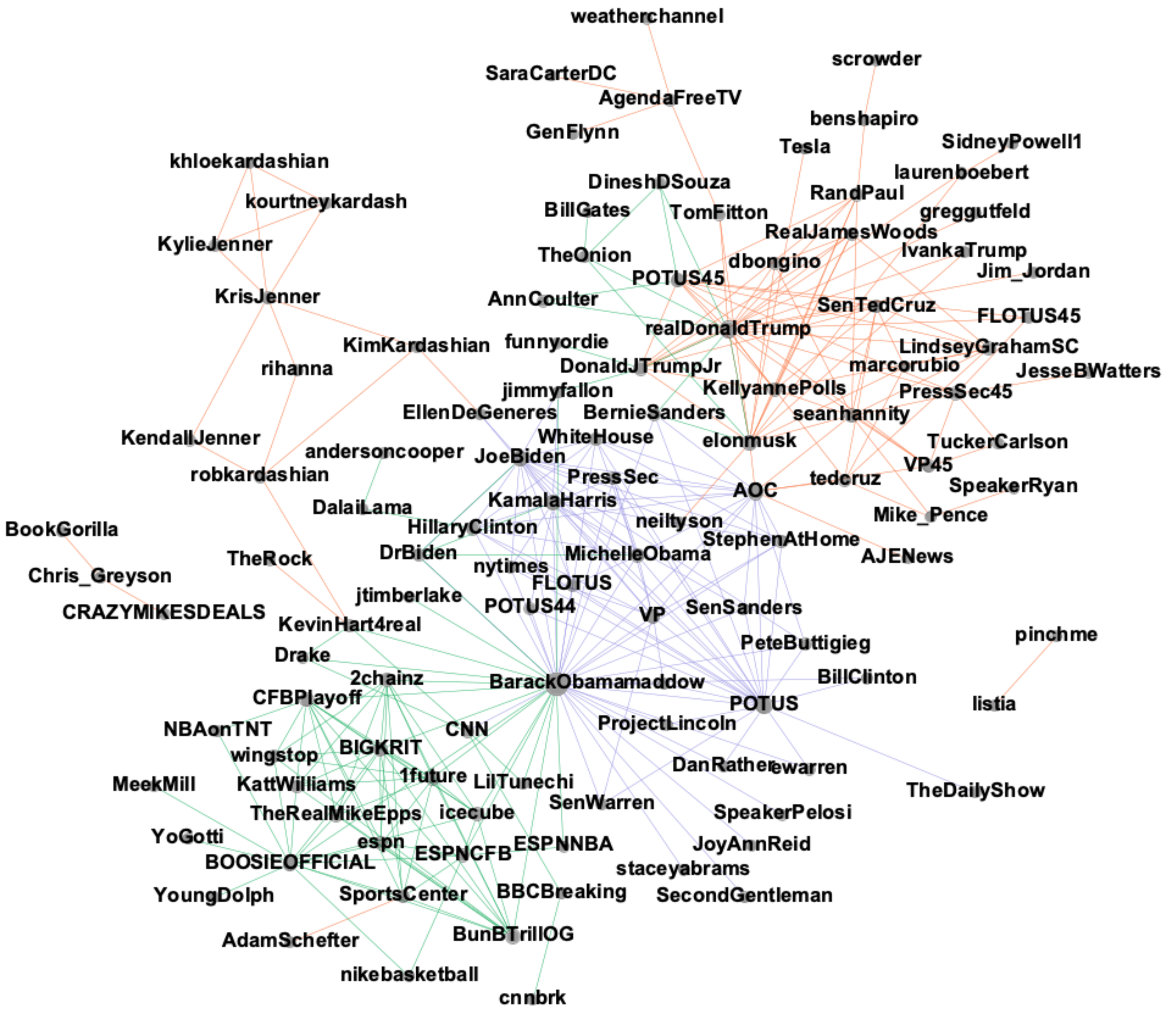

Supplementary Figure 1. Labeled network diagram of accounts followed by respondents on Twitter/X. Nodes are prominent accounts followed by consented Twitter/X respondents. An edge indicates that at least two respondents follow the pair of accounts. The thickness of the edge is based on the number of respondents following different pairs of nodes. The edge color shows the respondents vaccine status (purple = early adopter, green = late adopter, red = nonadopter).

Supplementary Table 4. Network Metrics (numbers in parentheses are confidence intervals from random network)

Modularity	0.176 (0.3717, 0.4183)
Average clustering coefficient	0.413 (0.0252,0.0682)
Number of connected components	3 (1, 3)
Largest diameter	9 (5, 7)

Supplementary Table 5. Supplementary List of Open-Ended Topics on Why Respondents Get Vaccinated

Topic	Percentage
science	2.70%
access	1.30%
incentive	0.70%
FDA	0.01%
others are	0.01%

Supplementary Table 6. Supplementary List of Open-Ended Topics Explaining Why Respondents Did Not Intend to Get Vaccinated

Topic	Percentage
distrust in science	4.60%
most people recover	3.90%
individual freedom	3.70%
FDA approval	3.30%
likely misinformation	2.40%
generally anti-vax	2%
does not prevent getting covid	0.90%
religion	0.90%
health issue	0.60%

Supplementary Table 7. Sample Topic Words for Getting Vaccinated Topics

**Top 5 Getting Vaccinated Topics**	**Words and Phrases Used to Identify Topic**
Personal Safety and health	age, death, diabetic, severity, hospitalization, cancer, asthma, alive, pregnancy
Stop spread/protect others	family, grandparents, kid, elderly, community, neighbors, spread, protect others, prevent transmitting
Work/job	employer, mandate, mandated, employment, industry, military, required, job
Travel	travel, traveling, cruise, trip, vacation, europe, fly
Normal life/easy option	concert, back to normal, wedding, attend, resume, stop wearing mask, go to events

Supplementary Table 8. Sample Topic Words for Not Getting Vaccinated Topics

**Top 5 Not Getting Vaccinated Topics**	**Words and Phrases Used to Identify Topic**
Too early	rushed, too new, too fast, too quick, too soon, untested, limited research, unknowns, not ready
Side effects	side effects, allergic, allergy, clots, anaphylaxis, bad reaction, negative effects, reactions
Trust	lack trust, sketchy, controversy, scam, conspiracy, politics, skeptical, distrust, don’t believe
Already had	already had, already covid, herd immunity, had disease, had covid, got covid
Vaccine safety	unsafe, not safe, safety issues, safeness, dangerous

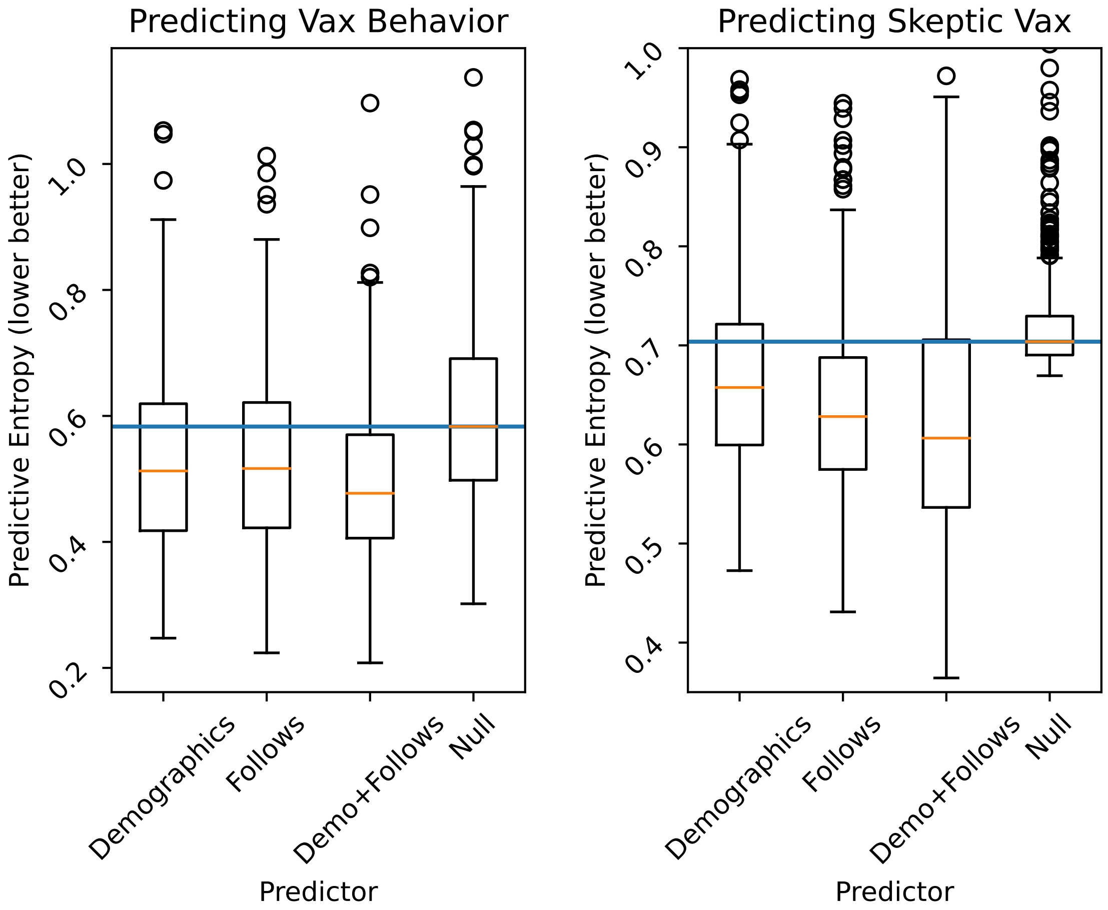

Supplementary Figure 2. Predictive study estimating vaccination status with various variables. *Left*: 3 class problem predicting Early Adopter, Vaccinated Skeptic and Persistent Skeptic based on demographic information, Twitter/X following information, and both. We find that follows and demographics provide similar predictions, and combining the two provides a very small improvement. *Right*: Among those not intending to vaccinate or unsure in Wave 0, we predict eventual vaccination status based on demographics, Twitter/X follows, and survey response topics. Twitter/X follows is most informative.

Survey Questions

Wave 0:

- For participants who chose “Not getting vaccinated” or “Unsure” in response to “Do you plan on getting vaccinated for the coronavirus once a vaccine is available to you?”
    - *“What are the main reasons you would not get a coronavirus vaccine?”*

Wave 1 and 2:

- For participants who selected that they were partially or fully vaccinated:
    - *“What were your main reasons for getting vaccinated?”*
- For participants who selected “No” or “Unsure” in response to “Do you plan on getting vaccinated for the coronavirus?”
    - *“What are the main reasons you would not get a coronavirus vaccine?”*

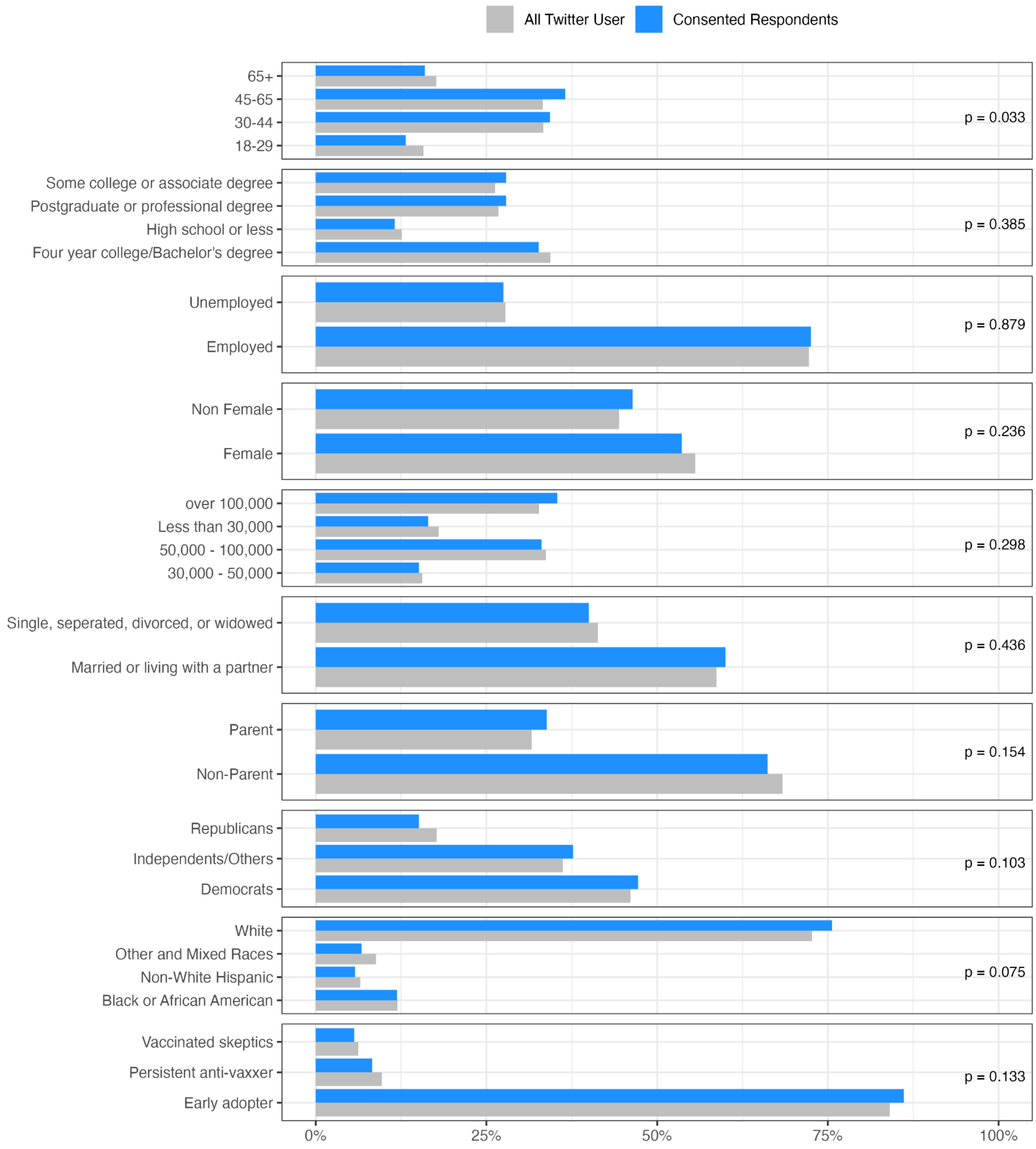

Supplementary Figure 3. Twitter Consent Check by Demographics and Vaccine Status. *The grey* bar indicates the proportions among all the Twitter users in the survey; the blue bars are the consented respondents who provide their Twitter handles. P-values, derived from the chi-squared test for equality of proportions, are presented for each category.

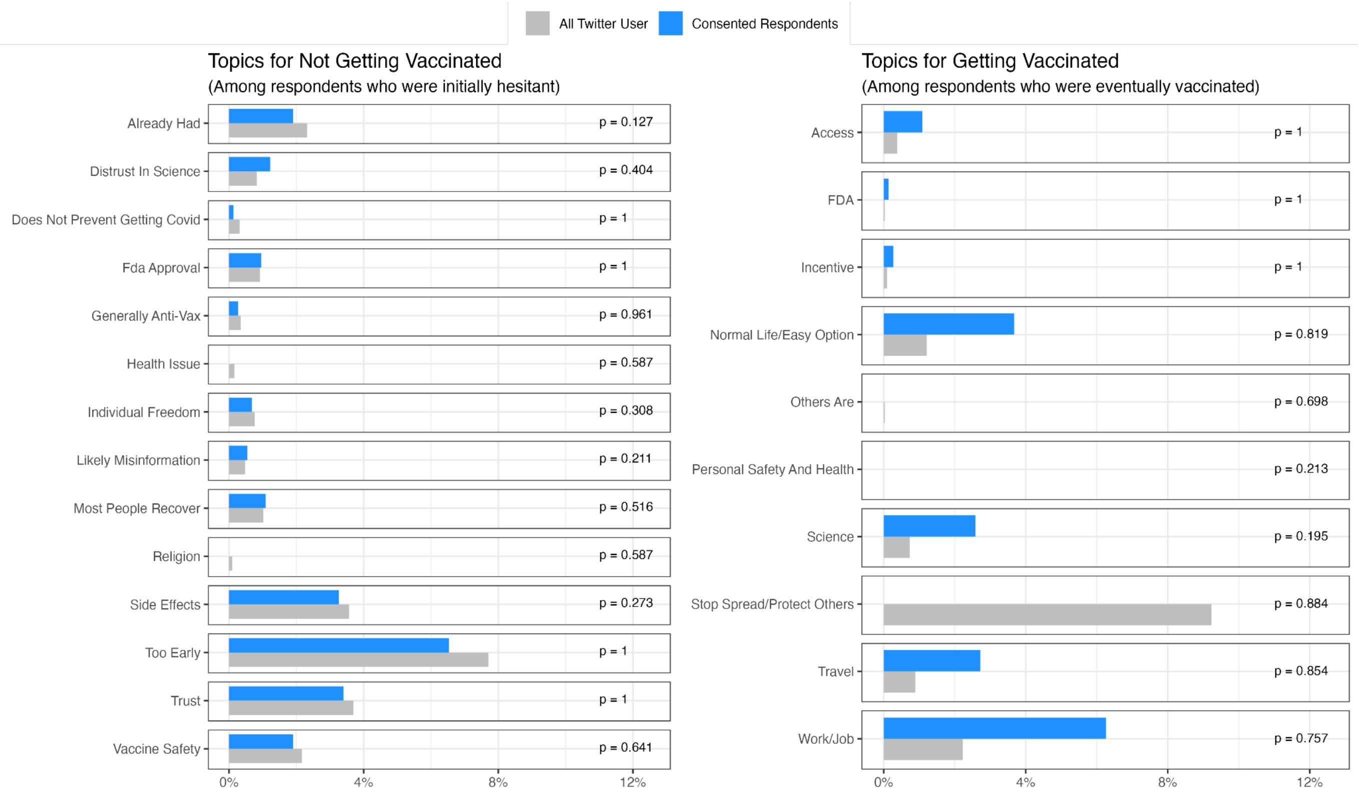

Supplementary Figure 4. Twitter Consent Check by Open-Ended Flags. *The grey* bar indicates the proportions among all the Twitter users in the survey; the blue bars are the consented respondents who provide their Twitter handles. P-values, derived from the chi-squared test for equality of proportions, are presented for each category.

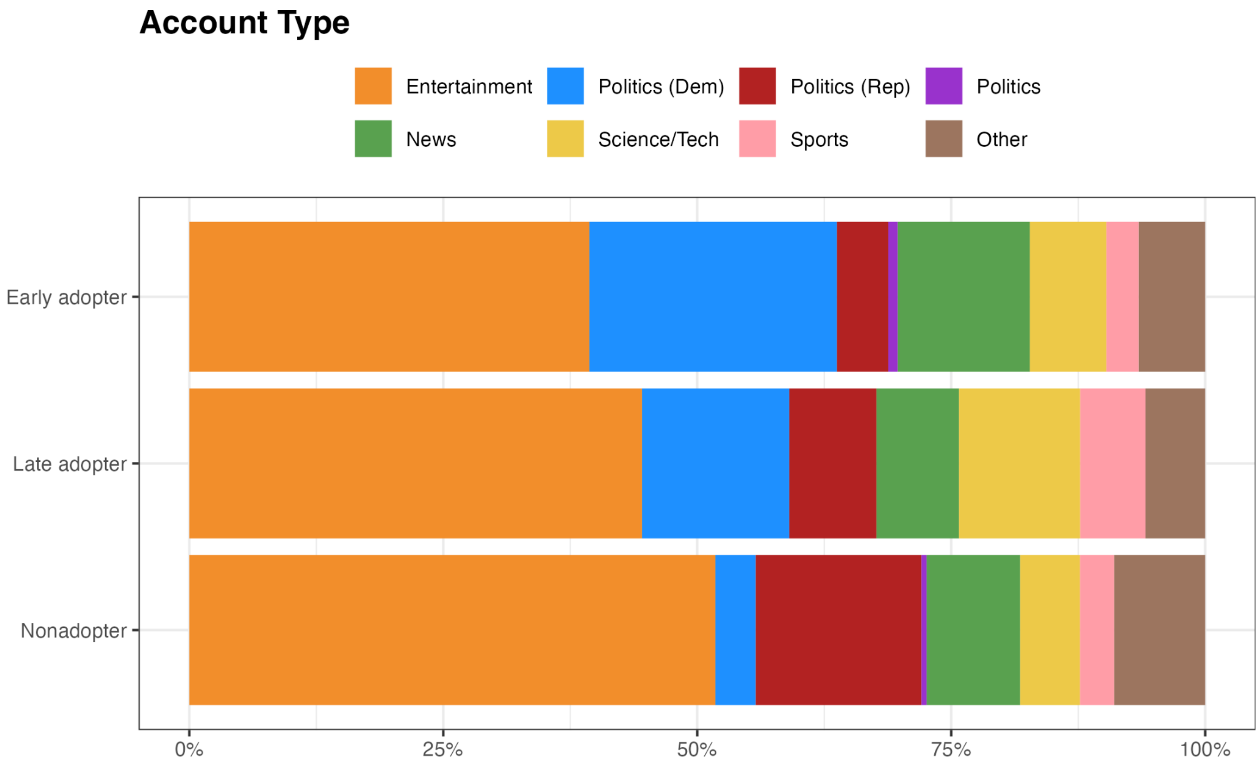

Supplementary Figure 5. Top 100 Followed Twitter/X Accounts by Account Types (% of consented Twitter/X respondents from each category). The x-axis shows the proportion of consented Twitter/X respondents in a specific group that follow different types of accounts. The colors of bars represent the different types of accounts.
